# Mechanical Property Changes in Breast Cancer Cells Induced by Stimulation with Macrophage Secretions in Vitro

**DOI:** 10.3390/mi10110738

**Published:** 2019-10-30

**Authors:** Hyonchol Kim, Kenta Ishibashi, Tomoko Okada, Chikashi Nakamura

**Affiliations:** 1 Biomedical Research Institute, National Institute of Advanced Industrial Science and Technology (AIST), 1-1-1 Higashi, Tsukuba, Ibaraki 305-8565, Japan; s189320z@st.go.tuat.ac.jp (K.I.); t.okada@aist.go.jp (T.O.); chikashi-nakamura@aist.go.jp (C.N.); 2 Graduate School of Engineering, Tokyo University of Agriculture and Technology, 2-24-16 Nakamachi, Koganei, Tokyo 184-8588, Japan

**Keywords:** intercellular adhesion strength, breast cancer cell, macrophage, secretion, mechanical property, atomic force microscopy

## Abstract

The contribution of secretions from tumor-associated macrophage (TAM)-like cells to the stimulation of mechanical property changes in murine breast cancer cells was studied using an in vitro model system. A murine breast cancer cell line (FP10SC2) was stimulated by adding macrophage (J774.2) cultivation medium containing stimulation molecules secreted from the macrophages, and changes in mechanical properties were compared before and after stimulation. As a result, cell elasticity decreased, degradation ability of the extracellular matrix increased, and the expression of plakoglobin was upregulated. These results indicate that cancer cell malignancy is upregulated by this stimulation. Moreover, changes in intercellular adhesion strengths between pairs of cancer cells were measured before and after stimulation using atomic force microscopy (AFM). The maximum force required to separate cells was increased by stimulation with the secreted factors. These results indicate the possibility that TAMs cause changes in the mechanical properties of cancer cells in tumor microenvironments, and in vitro measurements of mechanical property changes in cancer cells will be useful to study interactions between cells in tumor microenvironments.

## 1. Introduction

Tumor microenvironments are composed of various cells, including stromal cells, and play critical roles in tumor metastasis [1]. Tumor-associated macrophages (TAMs) in particular are considered to contribute to the upregulation of tumor malignancy [2,3,4], and pathological evidence has indicated that the abundance of TAMs strongly correlates with poor prognosis [5,6,7]. It is known that TAMs and cancer cells can stimulate each other through the secretion of molecules in the tumor microenvironment, as an example of a paracrine loop [3], and this interactive stimulation promotes tumor growth and metastasis. Recently, it was found that a portion of TAMs expressed programmed cell death protein 1 (PD-1), and a relationship between the expression of PD-1 on TAMs and advantageous phenotypes for tumor progression was indicated [8]. Despite the importance of interactive stimulation between TAMs and cancer cells for tumor development, the contribution of this stimulation to the regulation of cancer cell mechanical properties s remains poorly studied.

It is well known that the mechanical properties of cancer cells are strongly correlated with cancer cell malignancy. For example, the elasticity of cancer cells, which is usually quantified by measuring cells’ Young’s moduli, correlates with their metastatic abilities [9,10]. Atomic force microscopy (AFM) is a useful tool to measure the mechanical properties of cells, because the AFM tip can contact cell surfaces directly, and various cell mechanical parameters can be measured quantitatively, including elasticity and adhesive strength [10,11,12,13,14,15,16,17,18]. In our previous study, a method to measure intercellular adhesion strengths between two cells was suggested using an AFM chip with an attached micro-cup [19]. In this study, the contribution of stimulation by secretions from TAM-like macrophages to changes in the mechanical properties of breast cancer cells was studied using this method. Firstly, we established an in vitro model of the macrophage stimulation of cancer cells as a mimic of the stimulation with tumor-conditioned medium [20,21]. Mechanical properties such as intercellular adhesion strength and cell elasticity were measured quantitatively and compared before and after stimulation to evaluate how the stimulation contributed to the regulation of cancer cell malignancy.

## 2. Materials and Methods 

### 2.1. Cells and the in Vitro Model of Cancer Cell Stimulation

In this study, two cell lines were used. J774.2 cells consist of monocyte macrophages from a BALB/c mouse (DS Pharma Biomedical, Osaka, Japan). This cell line was originally established from ascites and solid tumor [22], and has TAM-like characteristics such as the expression of PD-1 (Supplementary Figure S1) [8]. The other cell line was FP10SC2, a mammary carcinoma from a BALB/c mouse (DS Pharma Biomedical, Osaka, Japan). This cell line was established from 4T1 (a breast cancer cell line) as a highly metastatic cell line [23]. Both cell lines were cultured in commercially available culture media (DMEM for J774.2 and RPMI-1640 for FP10SC2, Thermo Fisher Scientific, Waltham, MA, USA) containing 10% fetal bovine serum (FBS) and 50 U/mL penicillin plus 50 µg/mL streptomycin at 37 °C in 5% CO_2_ conditions.

As an in vitro model of cancer cell stimulation by secreted molecules from TAMs, the culture medium from J774.2 cell was added to the medium of FP10SC2 as follows. J774.2 was incubated in 2 mL of fresh culture medium with 2.5 × 10^5^ cells for 1 day. The medium was then collected, centrifuged in 200 × *g* for 3 min to remove debris, and the supernatant was used for stimulation (the obtained medium is hereafter referred to as macrophage-conditioned medium (MΦ-CM)). On the other hand, FP10SC2 cells were incubated with 5 × 10^4^ cells for 1 day, the medium was discarded, and MΦ-CM was added to the dish. The dish was incubated for 1 day to stimulate cancer cells with secretions contained in MΦ-CMs (these stimulated cells are hereafter referred to as macrophage-conditioned FP10SC2 (MC-FP10SC2)). As a negative control, culture medium of FP10SC2 cells was exchanged for 2 mL of fresh medium instead of the addition of MΦ-CM, and incubated in the same manner as for MC-FP10SC2 (these control cells are hereafter referred to as FP10SC2).

### 2.2. Measurements of Cell Elasticity

The elasticities of cancer cells (FP10SC2 and MC-FP10SC2) were evaluated quantitatively using Young’s modulus calculated from the approach period of the force curve in AFM measurements (Nanowizard II, JPK, Berlin, Germany). For these measurements, a microbead (10-μm diameter, JSR, Tokyo, Japan) attached to the AFM chip was used [24]. Conditions for the force measurements were as follows: *Z* scan size 30 μm, *Z* scan velocity 5 μm/s, and loading force 3 nN. Measurements were performed on a cell surface avoiding the cell nucleus. The measurement was performed once per cell and repeated on 60 individual cells before and after stimulation, and average values were calculated. Spring constants of the AFM cantilevers (DNP, Bruker, Billerica, MA, USA) were calibrated using the thermal fluctuation method, and the typical value was 0.06 N/m. Young’s modulus, *E*, was calculated using the following Hertzian contact model for a rigid spherical indenter and elastic substrate [25,26]:

(1) $$E=\frac{3F\left(1-\vartheta^{2}\right)}{4d^{\frac{3}{2}}r^{\frac{1}{2}}}$$

with the applied force *F*, Poisson’s ratio of the cell *ν*, indentation depth *d*, and radius of spherical indenter *r*. Here, the value of *ν* was 0.5 for all calculations [27,28,29]. For the calculation of *d*, a contact point between the AFM chip and the cell surface in each force curve was estimated as follows. Values of both average and standard deviation (SD) of the cantilever deflection at the initial 10% of the *Z* position in the approach period (i.e., the cantilever was far from the cell surface and not contacting at the position) were calculated, and a point at which repulsive deflection of the cantilever became larger than the value of the average plus 5 SD in the approach period was determined as the contact point of the force curve. The typical value of *d* in each force curve was within a range between 700 and 1200 nm. Then, *E* was calculated by fitting the approach curve between the contact point and the maximum loading point (i.e., the loading force was 3 nN at the point) with Equation (1) by using software (KaleidaGraph 4, Hulinks, Tokyo, Japan).

### 2.3. Measurements of Cell Invasiveness

Measurements of cell invasiveness were performed using a commercially available Boyden chamber system (Transwell, 8 µm pore diameter, Corning, Corning, NY, USA) as follows. Firstly, a mixture of extracellular matrix (Matrigel, Corning, Corning, NY, USA) was adjusted to 160 µg/mL in serum-free culture medium, added to the top chamber of the system, incubated for 3 h, and excess medium was removed. Next, MC-FP10SC2 cells were suspended in MΦ-CM at a concentration of 1 × 10^5^ /mL, and 200 µL of the suspension (2 × 10^4^ cells) was added to the top chamber of the system. The cells were incubated for 1 day in cell incubation conditions. After incubation, the top chamber was washed with phosphate-buffered saline (PBS), cells still on the top face of the chamber were discarded by scraping, and the remaining cells (i.e., cells adhering to the reverse face of the top chamber) were fixed with 4% paraformaldehyde in PBS for 10 min in room temperature. The cells were washed with PBS three times, and their nuclei were stained with 4 µM 4’,6-diamidino-2-phenylindole (DAPI, Dojindo, Japan) in PBS for 30 min at room temperature to make cell counting easy. After staining, the number of cells that migrated to the reverse face of the chamber with degradation of the extracellular matrix was counted. About 100 microscopic pictures were taken for an assay, a merged image under whole area of the top chamber was constructed, and numbers of cells were counted. The same experiments were repeated 10 times, and the average is presented. As a control, FP10SC2 cells were suspended in normal culture medium, seeded in the top chamber of the system, and the same measurement as for MC-FP10SC2 was performed.

### 2.4. Immunofluorescence Assays

Molecular expression levels of plakoglobin, desmoglein, E-cadherin, and claudin-4 were evaluated by immunofluorescence measurements as follows. Cancer cells (FP10SC2 and MC-FP10SC2) cultivated on glass-bottomed dishes (AGC Techno Glass, Shizuoka, Japan) with 5 × 10^4^ cells were fixed with 4% paraformaldehyde in PBS for 15 min at room temperature. The cells were washed with PBS and incubated with 2 µg/mL of the primary antibodies (rabbit anti-mouse plakoglobin, Abcam, Cambridge, UK; rabbit anti-mouse desmoglein, Bioss, Woburn, MA, USA; rabbit anti-mouse E-cadherin, Genetex, Owen, CA, USA; or rabbit anti-mouse claudin-4, Bioss, Woburn, MA, USA) in PBS containing 0.4% commercially available skim milk-based blocking reagent (Block Ace, DS Pharma Biomedical, Osaka, Japan) for 1 h at room temperature. After washing with PBS, 2 µg/mL of the secondary antibody (Alexa Fluor 568 donkey anti-rabbit IgG, Thermo Fisher, Waltham, MA, USA) in PBS containing 0.4% of the blocking reagent was applied to the dishes and reacted with the cancer cells for 1 h at room temperature. After washing with PBS, fluorescence observations were performed using an inverted optical microscope (IX-70, Olympus, Tokyo, Japan) combined with a cooled CCD camera (DP30BW, Olympus, Tokyo, Japan) using a 20× objective lens. The exposure time of the camera was set as 3 s. Fluorescence intensities of the obtained cell images were quantified using image processing software (Image J 1.6.0_24, National Institute of Health, Bethesda, MD, USA). In addition, distributions of F-actin in cancer cells were evaluated as follows. Cultivation and fixation of cancer cells were performed the same as for the immunofluorescence assays. After washing with PBS, 0.2% TritonX-100 containing 0.1% sodium dodecyl sulfate (SDS) in PBS was added to the culture dishes and incubated for 2 min for permeabilization. The cells were washed with PBS, 0.4% of the blocking reagent was applied, and they were incubated for 15 min. After washing with PBS, 30 nM rhodamine phalloidin (Thermo Fisher, Waltham, MA, USA) in PBS containing 0.4% of the blocking reagent was applied to the dishes and reacted with the cancer cells for 1 h at room temperature. After washing with PBS, fluorescence observations were performed with a 20× objective lens. Exposure time of the camera was set as 350 ms.

### 2.5. Measurements of Intercellular Adhesion Strengths by AFM

Measurements of the intercellular adhesion strengths between two cancer cells were performed using a cup-attached AFM chip, as reported previously [19]. The method to fabricate cup-shaped particles was also reported previously [30]. Briefly, polystyrene particles (10 µm diameter, JSR, Tokyo, Japan) were placed on a silicon substrate, and a 100-nm-thick nickel layer was formed on the particles by vapor deposition (VPC-260, ULVAC, Miyazaki, Japan). The polystyrene particles were removed in high-temperature conditions, and cup-shaped particles were obtained. The micro-cup was attached to the apex of an AFM cantilever with epoxy resin using a micromanipulator (MN-4 and MMO-202ND, Narishige, Tokyo, Japan). The fabricated AFM chip is hereafter referred to as the “cup-chip”.

Measurements of adhesion forces between two cancer cells were also performed in the same manner as reported previously [19]. The detailed experimental conditions in this study were as follows. For the pick-up of a cell by using the cup-chip, a cancer cell line (FP10SC2 or MC-FP10SC2) was detached from the bottom of a culture dish by treatment with 2.5 mg/mL trypsin and 380 µg/mL EDTA (Thermo Fisher Scientific, Waltham, MA, USA). A piece of polytetrafluoroethylene (PTFE)-coated glass substrate [29] was mounted on the bottom of another culture dish, on which the same cell line (FP10SC2 or MC-FP10SC2) was cultivated and the cells were still adhered, and the detached cell suspension was added to the PTFE-coated substrate. A cell was picked up using the cup-chip on the PTFE-coated substrate and approached the other cell adhered to the dish bottom, and force curve measurements were performed using an AFM. Conditions of force measurements were as follows: *Z* scan size 100 μm, *Z* scan velocity 5 μm/s, loading force 1 nN, and 5 s dwell time, maintaining a constant height of the cantilever at the sample. Measurements were performed on 69 MC-FP10SC2 cells and 89 FP10SC2 cells.

### 2.6. Data Analysis of AFM Measurements

The obtained force curves were analyzed using the software provided by the manufacturer (JPK Data Processing, JPK, Berlin, Germany). Firstly, a baseline force curve was defined as an average value of the force at a range of *Z* positions between 90 and 100 µm (i.e., far enough from the cell surface on the dish, and measured two cells that were completely separated) in the retraction period. Next, two parameters were calculated from force curves: the maximum force of separation in the retraction curve, and the separation work. The latter was calculated from an area of hysteresis surrounded between the retraction curve and the baseline in force–extension curves [11,17,18]. Separation work was normalized to the contact area, which was estimated based on a Hertzian contact model [25,26,31] and calculated from the approach period in each force curve.

## 3. Results

**Figure 1:**
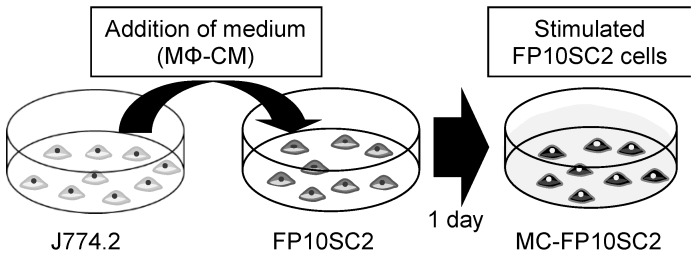
Schematic image of the in vitro stimulation model. Tumor-associated macrophage (TAM)-like macrophages (J774.2) were cultivated for 1 day, then the macrophage-conditioned culture medium (MΦ-CM) was collected and added to a culture dish on which cancer cells (FP10SC2) were growing. The cancer cells were cultured for 1 day to stimulate the cells with secretion molecules from the macrophages contained in the applied medium. After stimulation, the mechanical properties of the cancer cells were measured.

Figure 1 shows a schematic image of a cancer cell stimulated by secreted molecules from TAM-like macrophages in vitro as a mimic of stimulation with tumor-conditioned medium [20,21]. FP10SC2 cells were stimulated for 1 day by adding MΦ-CM containing molecules secreted from TAM-like macrophages to the culture dish. The term of the stimulation was also tried for 4 days; however, results were almost the same as for 1 day of stimulation. This indicates that the stimulation for 1 day is enough, and the following discussions were carried out using results for 1 day of stimulation. The stimulated FP10SC2 cells are referred to as MC-FP10SC2 in this study.

**Figure 2:**
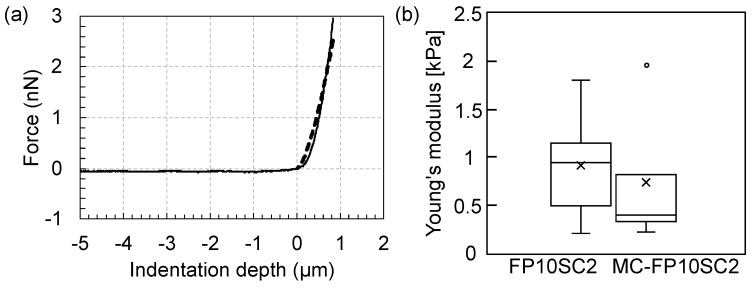
Changes in the Young’s modulus of cancer cells before and after stimulation with MΦ-CM. (**a**) Typical force–indentation curve (solid line) and a result of fitting based on Hertzian contact model (dashed line). *R*^2^ = 0.952. (**b**) Comparison of Young’s moduli before (FP10SC2) and after the stimulation (macrophage-conditioned FP10SC2—MC-FP10SC2). *P* < 0.01.

It was considered that cancer cell elasticity had a correlation with the ability of tumor metastasis, because softer cells could be expected to migrate through small gaps in tissues more smoothly, reach capillary vessels, and enter the blood circulation system [9,10]. In this study, changes in Young’s modulus before and after the stimulation with MΦ-CM were measured using a colloidal probe in the AFM. Figure 2 shows results of these measurements. The average Young’s modulus of MC-FP10SC2 decreased by 0.67 times compared with that before stimulation (i.e., FP10SC2), indicating that the elasticity of cancer cells decreased as a result of stimulation with MΦ-CM.

**Figure 3:**
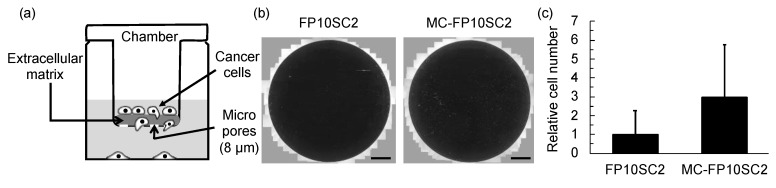
Evaluation of the degradation ability of extracellular matrices. (**a**) Schematic image of Boyden chamber system. (**b**) Typical images of migrated cancer cells with degradations of extracellular matrices. Bars: 1 mm. (**c**) Changes in the degradation abilities of extracellular matrices before and after stimulation with MΦ-CM measured using a Boyden chamber system. Values were normalized with an average value of FP10SC2. Standard deviations are indicated as error bars in the graph. *P* < 0.05.

In the process of tumor metastasis, cancer cells in a primary tumor migrate with the degradation of extracellular matrices to reach blood vessels. Therefore, the degradation ability of extracellular matrices is critically correlated with the invasive ability. Changes in the degradation ability of cancer cells induced by stimulation with MΦ-CM were evaluated using a Boyden chamber system. Figure 3 shows the results of an assay. The number of MC-FP10SC2 cells that migrated to the reverse face of the chamber with the degradation of extracellular matrices increased by 3.0 times on average compared with that of FP10SC2, indicating that the invasive ability of the cancer cells was increased by stimulation with MΦ-CM.

**Figure 4:**
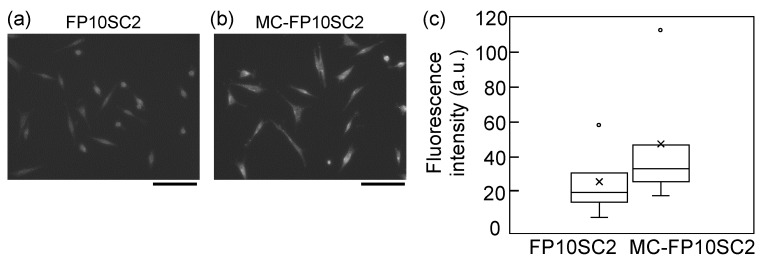
Immunofluorescence images of plakoglobin molecules on (**a**) FP10SC2 and (**b**) MC-FP10SC2 cells. Bars: 100 µm. (**c**) Comparison of relative fluorescence intensities between FP10SC2 and MC-FP10SC2 cells. *N* = 50 and *N* = 35 for FP10SC2 and MC-FP10SC2, respectively. *P* < 0.01.

It is known that highly malignant cancer cells form clusters in the blood (i.e., clusters of circulating tumor cells (CTC clusters)) [32,33,34]. A proportion of these cells were found to highly express plakoglobin, and high expression levels correlated with poor prognosis [32]. The change in expression levels of plakoglobins before and after the MΦ-CM stimulation was measured to evaluate the contribution of stimulation for the upregulation of plakoglobin expression. Figure 4 shows immunofluorescence images of plakoglobins before and after stimulation. Though distribution and localization were not significantly changed by the stimulation, the expression of plakoglobin in cancer cells clearly increased by stimulation with MΦ-CM.

**Figure 5:**
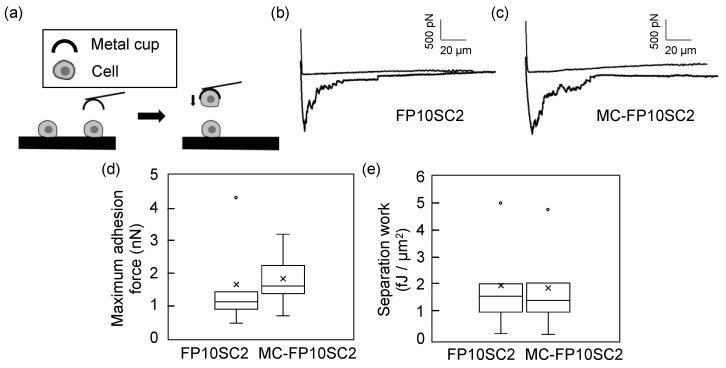
Measurements of changes in intercellular adhesion strengths. (**a**) Experimental design of the measurement system. A cancer cell was picked up using the cup-chip in the AFM, brought closer to another cancer cell, and force measurements were carried out. (**b**,**c**) Typical force curves obtained at the separation between two FP10SC2 cells (b) and two MC-FP10SC2 cells (c). (**d**,**e**) The boxplots of maximum adhesion forces (d) and separation work (e) of FP10SC2 and MC-FP10SC2 cells. *P* < 0.01 for (d).

Adhesion strengths between two MC-FP10SC2 cells were measured using the cup-chip in AFM measurements, and compared with that between two FP10SC2 cells. There is no serious damage done by the pickup of a cell using the cup-chip [30]. Figure 5 shows the results of the measurements. In this study, two parameters—maximum adhesion force and separation work—were used as evaluation parameters. The maximum adhesion force generally represented the maximum number of simultaneous bond breaks of adhesion molecules on the cell surface at the same time point, and separation work represented the total number of bond breaks in the contact area. In comparison with FP10SC2, the maximum adhesion force between two MC-FP10SC2 cells increased by 1.4 times on average (Figure 5a). On the other hand, separation work between two MC-FP10SC2 cells was almost the same as that before stimulation (Figure 5b). According to the former result, adhesion forces between two breast cancer cells were increased by stimulation with MΦ-CM.

## 4. Discussion

In this study, we focused on changes in the mechanical properties of breast cancer cells by stimulation with MΦ-CM. As a result, cancer cell elasticity decreased, invasive ability increased, and plakoglobin expression increased with stimulation. These results strongly suggest that malignancy in breast cancer cells increased with stimulation by MΦ-CM. MΦ-CM contained molecules secreted from TAM-like macrophages; therefore, it was likely that the measured changes resulted from the secretion of molecules by macrophages. The obtained results were consistent with the abundance of TAMs correlating with poor prognosis [5,6,7].

According to the results in Figure 5, the maximum adhesion force to separate cancer cells increased by 1.4 times, although there was no significant difference in separation work before and after stimulation with MΦ-CM. Separation work generally reflects the binding energy of two cells over their contact area, whereas work also includes deforming cell morphology and elongating cell membranes, such as a membrane tethers [16,18,35]. Strict discrimination of work between cell separation and deformation is difficult; therefore, the obtained results in Figure 5b contain the work of cell deformation. When the work of cell deformation was larger than that for two-cell separation, differences in work before and after stimulation were reduced even when the work to separate two cells was increased by stimulation. This may be why there was no significant difference in Figure 5b. On the other hand, maximum adhesion force generally reflects the strength of cell adhesion when the binding events occur uniformly over a contact area. Although various receptors are localized on the cell membrane, their size is nanoscopic and the size of the contact area in this study was on the scale of square micrometers. Therefore, it may be assumed that the biological properties of the cell membrane over the contact area were almost uniform in our experimental system, and the maximum adhesion force was a good index to compare adhesion strengths between two cells.

As shown in Figure 5, intercellular adhesion forces increased by stimulation with MΦ-CM. We evaluated changes in the expression levels of E-cadherin, desmoglein, and claudin-4, which are typical cell-adhesion-related receptors, depending on stimulation with MΦ-CM to find key mediators of the increase in adhesion force (Supplementary Figure S2). However, there were no clear differences in expression in these three receptors between FP10SC2 and MC-FP10SC2. In addition, distributions of F-actins in the two cancer cells were observed with fluorescence measurements (Supplementary Figure S3); however, there were also no clear differences between FP10SC2 and MC-FP10SC2. These results indicate that the increase in intercellular adhesion force induced by stimulation with MΦ-CM may be mediated by molecules other than these receptors.

## 5. Conclusions

In conclusion, we measured changes in the mechanical properties of breast cancer cells before and after stimulation with MΦ-CM in vitro. As a result, cell elasticity decreased, invasive ability increased, and plakoglobin expression was upregulated, strongly suggesting that cancer cell malignancy was increased by this stimulation. In addition, intercellular adhesion forces between the cancer cells increased with stimulation. Taken together, these results suggest that cancer cell malignancy was upregulated by TAM-like macrophage stimulation.

## Supplementary Materials

The following are available online at https://www.mdpi.com/2072-666X/10/11/738/s1, Figure S1: Immunofluorescence images of PD-1 expression on J774.2 (a) and MCF-7 (b, negative control) cells. Bright field (BF, left) and fluorescence (FL, right) images are shown. Bars: 50 μm. (c) Comparison of fluorescence intensities between J774.2 and MCF-7 cells. N = 62 for J774.2 and N = 57 for MCF-7. Figure S2: Immunofluorescence images of E-cadherin (a), desmoglein (b), and claudin-4 (c) molecules on FP10SC2 (left) and MC-FP10SC2 (right) cells. Bars: 100 μm. (d) Comparison of relative fluorescence intensities between FP10SC2 and MC-FP10SC2 cells for the three molecules. Values of FP10SC2 cells were used the standard for comparison. N = 38 and N = 43, N = 46 and N = 52, and N = 109 and N = 105 for FP10SC2 and MC-FP10SC2 cells for E-cadherin, desmoglein, and claudin-4, respectively. Figure S3: Fluorescence images of F-actin on FP10SC2 (left) and MC-FP10SC2 (right) cells. Bars, 100 μm.

## References

[1] Werb Z., Lu P. (2015). The role of stroma in tumor development. Cancer J..

[2] Aras S., Zaidi M.R. (2017). TAMeless traitors: macrophages in cancer progression and metastasis. Br. J. Cancer.

[3] Condeelis J., Pollard J.W. (2006). Macrophages: Obligate partners for tumor cell migration, invasion, and metastasis. Cell.

[4] Qian B.Z., Pollard J.W. (2010). Macrophage diversity enhances tumor progression and metastasis. Cell.

[5] Heusinkveld M., van der Burg S.H. (2011). Identification and manipulation of tumor associated macrophages in human cancers. J. Transl. Med..

[6] Komohara Y., Jinushi M., Takeya M. (2014). Clinical significance of macrophage heterogeneity in human malignant tumors. Cancer Sci..

[7] Komohara Y., Niino D., Ohnishi K., Ohshima K., Takeya M. (2015). Role of tumor-associated macrophages in hematological malignancies. Pathol. Int..

[8] Gordon S.R., Aute R.L.M., Dulken B.W., Hutter G., George B.M., Ccracken M.N.M., Gupta R., Tsai J.M., Sinha R., Corey D. (2017). PD-1 expression by tumour-associated macrophages inhibits phagocytosis and tumour immunity. Nature.

[9] Cross S.E., Jin Y.S., Rao J., Gimzewski J.K. (2007). Nanomechanical analysis of cells from cancer patients. Nat. Nanotechnol..

[10] Luo Q., Kuang D., Zhang B., Song G. (2016). Cell stiffness determined by atomic force microscopy and its correlation with cell motility. Biochim. Biophys. Acta.

[11] Kim H., Arakawa H., Osada T., Ikai A. (2002). Quantification of fibronectin and cell surface interactions by AFM. Colloids Surf. B.

[12] Kim H., Arakawa H., Osada T., Ikai A. (2003). Quantification of cell adhesion force with AFM: Distribution of vitronectin receptors on a living MC3T3-E1 cell. Ultramicroscopy.

[13] Kim H., Arakawa H., Hatae N., Sugimoto Y., Matsumoto O., Osada T., Ichikawa A., Ikai A. (2006). Quantification of the number of EP3 receptors on a living CHO cell surface by the AFM. Ultramicroscopy.

[14] Benoit M., Gabriel D., Gerisch G., Gaub H.E. (2000). Discrete interactions in cell adhesion measured by single-molecule force spectroscopy. Nat. Cell Biol..

[15] Chen A., Moy V.T. (2000). Cross-linking of cell surface receptors enhances cooperativity of molecular adhesion. Biophys. J..

[16] Puech P.H., Poole K., Knebel D., Muller D.J. (2006). A new technical approach to quantify cell-cell adhesion forces by AFM. Ultramicroscopy.

[17] Helenius J., Heisenberg C.P., Gaub H.E., Muller D.J. (2008). Single-cell force spectroscopy. J. Cell Sci..

[18] Muller D.J., Helenius J., Alsteens D., Dufrene Y.F. (2009). Force probing surfaces of living cells to molecular resolution. Nat. Chem. Biol..

[19] Kim H., Yamagishi A., Imaizumi M., Onomura Y., Nagasaki A., Miyagi Y., Okada T., Nakamura C. (2017). Quantitative measurements of intercellular adhesion between a macrophage and cancer cells using a cup-attached AFM chip. Colloids Surf. B.

[20] Chattopadhyay S., Roy S. (2019). Antigen conjugated nanoparticles reprogrammed the tumor-conditioned macrophages toward pro-immunogenic type through regulation of NADPH oxidase and p38MAPK. Cytokine.

[21] Solinas G., Schiarea S., Liguori M., Fabbri M., Pesce S., Zammataro L., Pasqualini F., Nebuloni M., Chiabrando C., Mantovani A. (2010). Tumor-conditioned macrophages secrete migration-stimulating factor: a new marker for M2-polarization, influencing tumor cell motility. J. Immunol..

[22] Ralph P., Prichard J., Cohn M. (1975). Reticulum cell sarcoma: an effector cell in antibody-dependent cell-mediated immunity. J. Immunol..

[23] Okada T., Kurabayashi A., Akimitsu N., Furihata M. (2017). Expression of cadherin-17 promotes metastasis in a highly bone marrow metastatic murine breast cancer model. Biomed Res. Int..

[24] Ducker W.A., Senden T.J., Pashley R.M. (1991). Direct measurement of colloidal forces using an atomic force microscope. Nature.

[25] Hertz H. (1881). Ueber die Beruhrung fester eleatischer Korper. J. Reine Angew. Math..

[26] Sneddon I.N. (1965). The relation between load and penetration in the axisymmetric boussinesq problem for a punch of arbitrary profile. Int. J. Engng. Sci..

[27] Haga H., Sasaki S., Kawabata K., Ito E., Ushiki T., Sambongi T. (2000). Elasticity mapping of living fibroblasts by AFM and immunofluorescence observation of the cytoskeleton. Ultramicroscopy.

[28] Tanaka A., Fujii Y., Kasai N., Okajima T., Nakashima H. (2018). Regulation of neuritogenesis in hippocampal neurons using stiffness of extracellular microenvironment. Plos One.

[29] Kim H., Ishibashi K., Matsuo K., Kira A., Onomura Y., Okada T., Nakamura C. (2018). Adhesion strength of a living cell to various substrates measured using a cup-attached atomic force microscopy chip. Jpn. J. Appl. Phys..

[30] Kim H., Terazono H., Takei H., Yasuda K. (2014). Cup-shaped superparamagnetic hemispheres for size-selective cell filtration. Sci. Rep..

[31] Fischer-Cripps A.C. (1999). The hertzian contact surface. J. Mater. Sci..

[32] Aceto N., Bardia A., Miyamoto D.T., Donaldson M.C., Wittner B.S., Spencer J.A., Yu M., Pely A., Engstrom A., Zhu H. (2014). Circulating tumor cell clusters are oligoclonal precursors of breast cancer metastasis. Cell.

[33] Kim H., Terazono H., Nakamura Y., Sakai K., Hattori A., Odaka M., Girault M., Arao T., Nishio K., Miyagi Y. (2014). Development of on-chip multi-imaging flow cytometry for identification of imaging biomarkers of clustered circulating tumor cells. Plos One.

[34] Hosokawa M., Kenmotsu H., Koh Y., Yoshino T., Yoshikawa T., Naito T., Takahashi T., Murakami H., Nakamura Y., Tsuya A. (2013). Size-based isolation of circulating tumor cells in lung cancer patients using a microcavity array system. Plos One.

[35] El-Kirat-Chatel S., Dufrene Y.F. (2016). Nanoscale adhesion forces between the fungal pathogen Candida albicans and macrophages. Nanoscale Horiz..

