# Supplementary material for: Mechanical Property Changes in Breast Cancer Cells Induced by Stimulation with Macrophage Secretions in Vitro
Source: Micromachines (Basel). 2019 Oct 30;10(11):738. doi: 10.3390/mi10110738 (PMC6915679; doi:10.3390/mi10110738)
Supplement: Supplementary file 1 [file micromachines-10-00738-s001.pdf]

micromachines

Supplementary Materials: Mechanical Property Changes in Breast Cancer Cells Induced by Stimulation with Macrophage Secretions in Vitro

Hyonchol Kim [1,2,#,*], Kenta Ishibashi [1,2,#], Tomoko Okada [1] and Chikashi Nakamura [1,2]

[1] Biomedical Research Institute, National Institute of Advanced Industrial Science and Technology (AIST), 1-1-1 Higashi, Tsukuba, Ibaraki 305-8565, Japan; t.okada@aist.go.jp (T.O.); chikashinakamura@aist.go.jp (C.N.)

[2] Graduate School of Engineering, Tokyo University of Agriculture and Technology, 2-24-16 Nakamachi, Koganei, Tokyo 184-8588, Japan; s189320z@st.go.tuat.ac.jp (K.I.)

* Correspondence: kim-hc@aist.go.jp; Tel.: +81-29-861-9392 (H.K.)

# These authors contributed equally to this work.

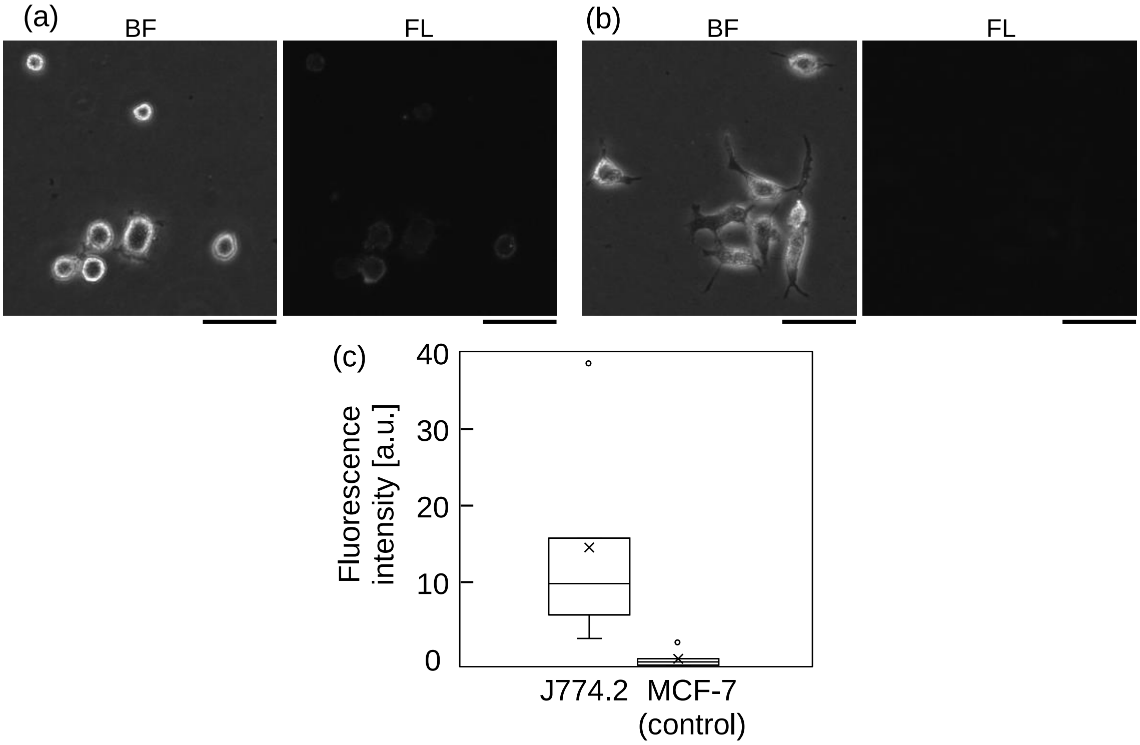

Figure S1. Images of PD-1 expression on J774.2 (**a**) and MCF-7 (**b**, negative control) cells. Bright field (BF, left) and fluorescence (FL, right) images are shown. Bars: 50 µm. (**c**) Comparison of fluorescence intensities between J774.2 and MCF-7 cells. *N* = 62 for J774.2 and *N* = 57 for MCF-7.

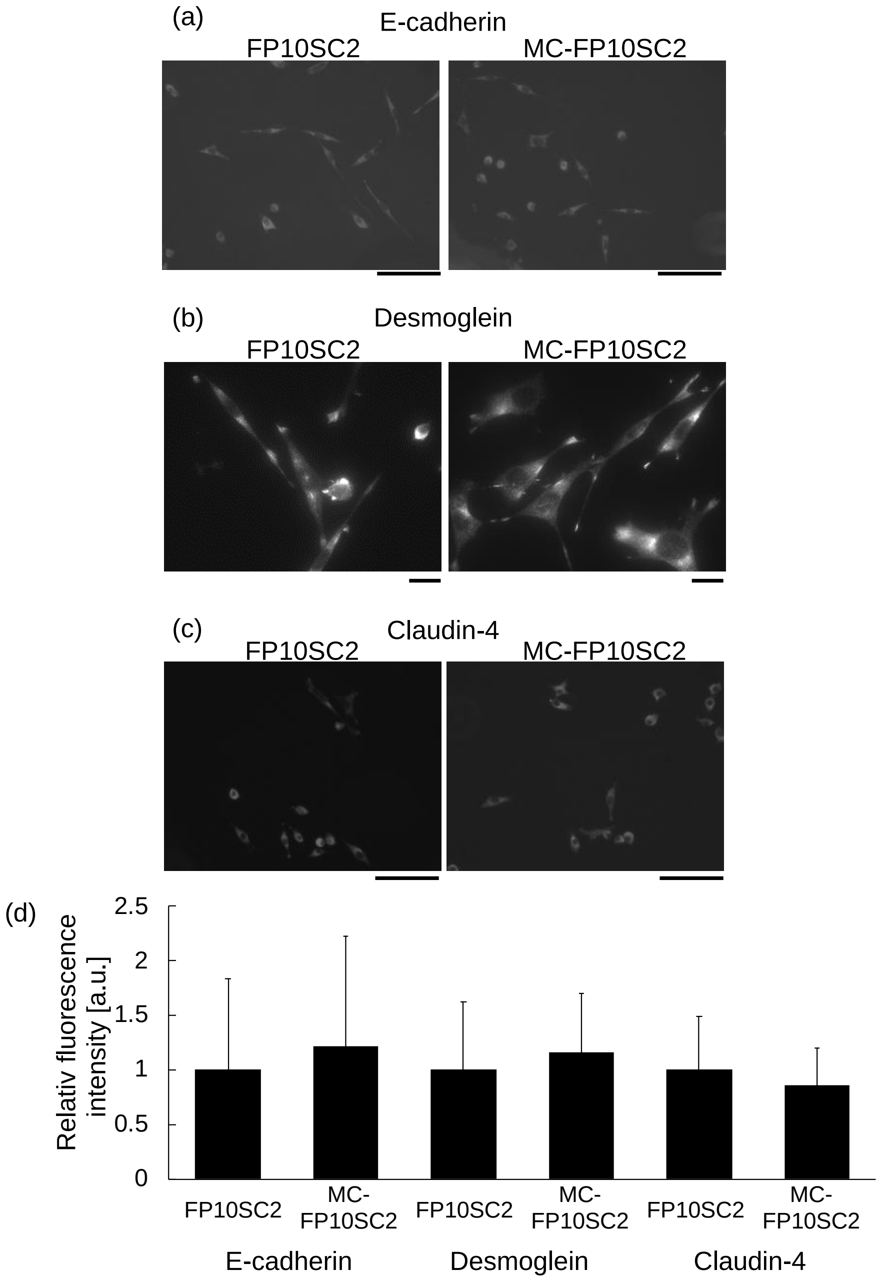

Figure S2. Images of E-cadherin (**a**), desmoglein (**b**), and claudin-4 (**c**) molecules on FP10SC2 (left) and MC-FP10SC2 (right) cells. Bars: 100 μm. (**d**) Comparison of relative fluorescence intensities between FP10SC2 and MC-FP10SC2 cells for the three molecules. Values of FP10SC2 cells were used the standard for comparison. N = 38 and N = 43, N = 46 and N = 52, and N = 109 and N = 105 for FP10SC2 and MC-FP10SC2 cells for E-cadherin, desmoglein, and claudin-4, respectively.

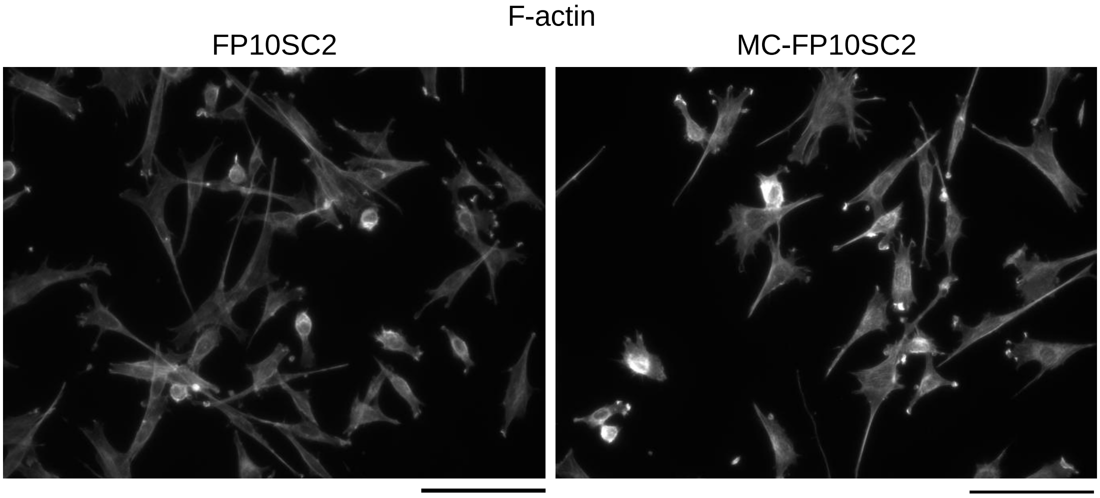

Figure S3. Images of F-actin on FP10SC2 (left) and MC-FP10SC2 (right) cells. Bars: 100 μm.
